# Methylation of *L1RE1*, *RARB*, and *RASSF1* function as possible biomarkers for the differential diagnosis of lung cancer

**DOI:** 10.1371/journal.pone.0195716

**Published:** 2018-05-31

**Authors:** R. F. H. Walter, P. Rozynek, S. Casjens, R. Werner, F. D. Mairinger, E. J. M. Speel, A. Zur Hausen, S. Meier, J. Wohlschlaeger, D. Theegarten, T. Behrens, K. W. Schmid, T. Brüning, G. Johnen

**Affiliations:** 1 Ruhrlandklinik, West German Lung Center, University Hospital Essen, University of Duisburg-Essen, Essen, Germany; 2 Institute of Pathology, University Hospital Essen, University of Duisburg-Essen, Essen, Germany; 3 Institute for Prevention and Occupational Medicine of the German Social Accident Insurance, Institute of the Ruhr-University Bochum (IPA), Bochum, Germany; 4 Department of Pathology, GROW-School for Oncology & Developmental Biology, Maastricht University Medical Center, Maastricht, the Netherlands; West Virginia University, UNITED STATES

## Abstract

**Background:**

Lung cancer is the major cause of cancer-related deaths worldwide. Differential diagnosis can be difficult, especially when only small samples are available. Epigenetic changes are frequently tissue-specific events in carcinogenesis and hence may serve as diagnostic biomarkers.

**Material and methods:**

138 representative formalin-fixed, paraffin-embedded (FFPE) tissues (116 lung cancer cases and 22 benign controls) were used for targeted DNA methylation analysis via pyrosequencing of ten literature-derived methylation markers (*APC*, *CDH1*, *CDKN2A*, *EFEMP1*, *FHIT*, *L1RE1*, *MGMT*, *PTEN*, *RARB*, and *RASSF1*). Methylation levels were analyzed with the Classification and Regression Tree Algorithm (CART), Conditional Interference Trees (ctree) and ROC. Validation was performed with additional 27 lung cancer cases and 38 benign controls. TCGA data for 282 lung cancer cases was included in the analysis.

**Results:**

CART and ctree analysis identified the combination of *L1RE1* and *RARB* as well as *L1RE1* and *RASSF1* as independent methylation markers with high discriminative power between tumor and benign tissue (for each combination, 91% specificity and 100% sensitivity). *L1RE1* methylation associated significantly with tumor type and grade (p<0.001) with highest methylation in the control group. The opposite was found for *RARB* (p<0.001). *RASSF1* methylation increased with tumor type and grade (p<0.001) with strongest methylation in neuroendocrine tumors (NET).

**Conclusion:**

Hypomethylation of *L1RE1* is frequent in tumors compared to benign controls and associates with higher grade, whereas increasing methylation of *RARB* is an independent marker for tumors and higher grade. *RASSF1* hypermethylation was frequent in tumors and most prominent in NET making it an auxiliary marker for separation of NSCLC and NET. *L1RE1* in combination with either *RARB* or *RASSF1* could function as biomarkers for separating lung cancer and non-cancerous tissue and could be useful for samples of limited size such as biopsies.

## Introduction

Lung cancer remains the number one cancer disease and main cause of cancer-related deaths worldwide. Differential diagnosis of the histological subtypes can be challenging, particularly when small biopsies or cytological specimens are available only. Biomarkers are helpful tools to improve classical pathologic-anatomical examinations and may even be used in body fluids.

DNA methylation of promoter regions is an important transcriptional regulator and its deregulation is associated with human diseases, especially cancer. In cancer, a global hypomethylation of the cancer cell genome is prominent, but certain genes, particularly tumor suppressor genes, present with *de novo* methylation leading to transcriptional inactivation. Furthermore, aberrant methylation is an early event in carcinogenesis and considered as one of the possible alterations that underlie the hallmarks of cancer [1–3]. Therefore, gene-specific methylation changes and their assessment were considered as molecular markers for risk stratification, tumor detection, and as therapy targets [2, 4]. *RARB* and *RASSF1* are important tumor suppressors and loss of expression is associated with a growth advantage and immortalization of tumor cells [5, 6]. Hypermethylation of tumor suppressor genes such as *CDKN2A*, *FHIT*, and *RARB* were reported to correlate with prognosis in adenocarcinomas [2]. *RASSF1* hypermethylation is another frequent event in lung cancer affecting approximately 60% of adenocarcinomas (ADC) and up to 100% of small cell lung carcinomas (SCLC) [7]. In contrast, hypomethylation of *EFEMP1* was identified as independent prognostic marker of non-small cell lung carcinoma (NSCLC) [2]. Another potential methylation biomarker is *L1RE1* (also known as *LINE1*), which is an autonomous transposable element in mammals and presents with hypomethylation in several cancers [8, 9]. Additionally, *L1RE1* represents approximately 17% of the human genome making it a global methylation marker [9]. Hypomethylation of *L1RE1* associates with stronger transcriptional activity, higher retrotransposition and hence genetic instability [9]. Several methylation and deacetylation inhibitors exist, which can reactivate gene expression by reversing the tumor-associated epigenetic alterations [5] indicating a potential clinical use of these drugs.

In the daily routine of modern pathology, formalin-fixed, paraffin-embedded tissue (FFPE) is the main source for molecular biological investigations. The fixation process modifies DNA leading to cross-links, degradations, and base-alterations. Daugaard et al. showed that DNA degradation has a minor impact on the results obtained when using methylation assessment methods that are mainly based on PCR [1]. Therefore, we conducted a retrospective analysis of 138 human FFPE lung samples, using pyrosequencing to screen for ten methylation markers and evaluated their ability to discriminate benign from tumor samples (exploratory dataset). To confirm our findings, we analyzed additional 65 FFPE lung samples (validation dataset). In addition, 282 lung cancer cases from the TCGA database were analyzed.

## Material and methods

Approximately 20 representative specimens of each tissue entity (21 typical (TC) and 17 atypical carcinoid tumors (AC), 19 large cell neuroendocrine carcinomas (LCNEC) and 21 small-cell lung cancer (SCLC), 23 adenocarcinoma (ADC) and 15 squamous cell carcinomas (SQCC), as well as 22 benign samples from patients with pneumothorax) were used for DNA methylation analysis. Samples of SCLC and benign cases were mainly obtained by biopsy, the other samples mainly by surgery. Patients that received chemotherapy before resection of tumor tissue specimens were excluded. The initial diagnosis was confirmed by two experienced pathologists (JW, DT). Additional inclusion criteria were sufficient tumor material and minimal contamination by benign and stromal cells. Specimens were drawn from the tumor bank at the Institute of Pathology, University Hospital Essen (Germany) from 2005 till 2012. TNM staging is based on the guidelines of the *2004 WHO Classification Of Tumors* [10] and clinical data were obtained from the patients’ records. For the benign samples (pneumothoraces) no clinical data was collected. The study was conducted retrospectively to identify methylation-based biomarkers. The study was approved by the ethical committee of the University Hospital Essen (ID: 13-5382-BO). The investigation conforms to the principles outlined in the declaration of Helsinki. Clinicopathological parameters are summarized in Table 1 A validation of the results was performed by analyzing additional 38 benign samples and 27 lung cancer cases (14 NSCLC; 13 NET; validation cohort). Additionally, 282 lung cancer cases from TCGA were analyzed (178 ADC and 104 SQCC). The data is accessible via http://cancergenome.nih.gov/. The results—published or shown—here are in whole or part based upon data generated by the TCGA Research Network: (TCGA-LUSC) [11] and are stored under http://doi.org/10.7937/K9/TCIA.2016.TYGKKFMQ (Kirk, S., Lee, Y., Kumar, P., Filippini, J., Albertina, B., Watson, M., Lemmerman, J. (2016). Radiology Data from The Cancer Genome Atlas Lung Squamous Cell Carcinoma [TCGA-LUSC] collection. The Cancer Imaging Archive.) and http://doi.org/10.7937/K9/TCIA.2016.JGNIHEP5 (Albertina, B., Watson, M., Holback, C., Jarosz, R., Kirk, S., Lee, Y., Lemmerman, J. (2016). Radiology Data from The Cancer Genome Atlas Lung Adenocarcinoma [TCGA-LUAD] collection. The Cancer Imaging Archive) [11]. TCGA data for benign lung or NET was not available with respect to methylation analysis.

**Table 1:** Investigated entities and clinicopathological parameters (exploratory dataset).

	Patients	Gender			Age at Diagnosis	Stage					Grade				
	N	Males	Females	Unknown	Age in years (mean)	pT1	pT2	pT3	pT4	NA	G1	G2	G3	G4	NA
**Benign**	22	NA	NA	22	NA	-	-	-	-	-	-	-	-	-	-
**Lung Cancer**	116	64	47	5	61.90	50	30	7	2	27	33	29	27	9	18
**TC**	21	7	13	1	56.03	15	5	0	0	1	19	0	0	0	2
**AC**	17	6	11	0	57.45	12	4	0	0	1	12	4	0	0	1
**LCNEC**	19	12	7	0	65.04	10	6	1	1	1	0	4	13	0	2
**SCLC**	21	16	4	1	43.53	2	1	2	1	15	0	0	8	9	4
**ADC**	23	12	9	2	65.60	8	10	2	0	3	2	15	4	0	2
**SQCC**	15	11	3	1	70.35	3	4	2	0	6	0	6	2	0	7

NA: not available

### DNA extraction

Five paraffin sections with a thickness of 10 μm per sample were collected. Deparaffination was performed by vortexing the slices in 1 ml Roti-Histol for one minute and subsequent centrifugation. The supernatant was discarded and the whole step was repeated. After that, the tissue was washed twice with 96% ethanol (vortexed for one minute, short centrifugation and removal of the supernatant). After the last centrifugation, the tissue pellet was dried in a SpeedVac for approximately 3 minutes. Starting with a Proteinase K incubation in ATL buffer at 56°C overnight, the DNA was extracted with the QIAamp DNA FFPE Tissue Kit (Qiagen, Hilden, Germany) on a QIAcube, following the manufacturer’s recommendations.

### DNA methylation analysis

Prior to the DNA methylation analysis itself, each DNA sample was bisulfite converted, using the EpiTect Bisulfite Kit (Qiagen) according to the manufacturer’s instructions, with 500 ng of DNA for each reaction. The thermal-cycling conditions were extended by a denaturation step of 5 min at 95°C followed by 2 h at 60°C before the final step (hold at 20°C).

The gene targets of the methylation assays comprised of *APC*, *CDH1*, *CDKN2A*, *EFEMP1*, *FHIT*, *L1RE1*, *MGMT*, *PTEN*, *RARB*, and *RASSF1*, which were chosen as potential biomarkers based on recent literature [2, 6–9, 12, 13]. The individual primer sets and PCR conditions are listed in Tables 2 and 3, respectively. All amplifications were performed using the PyroMark PCR Kit (Qiagen) with 3 μl of bisulfite-converted DNA on a Veriti Thermal Cycler (Thermo Fisher Scientific, Foster City, USA) following standard protocols. Proper PCR products were verified by gel electrophoresis prior to the sequencing reactions. DNA methylation analysis was performed by pyrosequencing on a PyroMark Q96 ID pyrosequencer (Qiagen) using the PyroMark Q96 Vacuum Prep Workstation and the PyroMark Gold Q96 Reagents (Qiagen) according to the manufacturer’s instructions [14].

**Table 2:** Primers for amplification and pyrosequencing of gene promoter regions.

Assay	Forward Primer (5'->3')	Reverse Primer (5'->3')	Sequencing primer (5'->3')
APC	GGGGTTAGGGTTAGGTAGGTT	BIO-ACTACACCAATACAACCACATATC	GAGAGAAGTAGTTGTGTAAT
CDH1	ATTTTAGTAATTTTAGGTTAGAGGGTTA	BIO-ACCACAACCAATCAACAAC	ATTTTAGGTTAGAGGGTTAT
EFEMP1	ATTTTATAGGAGTTGGTTAGAAGTT	BIO-ACAAAAAAATAAAATCCCCTTTCTTAACA	TTGGTTAGAAGTTGGG
FHIT	GGGGAGGTAAGTTTAAGTGGAATATTG	BIO-ATCCCCACCCTAAAACCCT	TAAGTGGAATATTGTTTTTGGG
L1RE1	TTTTGAGTTAGGTGTGGGATATA	BIO-AAAATCAAAAAATTCCCTTTC	AGTTAGGTGTGGGATATAGT
MGMT	GGATATGTTGGGATAGTT	BIO-CCCAAACACTCACCA	GGGATAGTTAGAGTTTTTAGAA
CDKN2A (P14)	TGTTTATTTTTGGTGTTAAAGG	BIO-CTAACTCCTCAATAACATCAACAC	GGTTTTTGGTGATTTTT
PTEN	GGTGATGTGGTAGGATTTTTTAT	BIO-CAAACTTCCATCATAACTACAACTT	ATGTGGTAGGATTTTTTATG
RARB	TGTTAAAGGGGGGATTAGAAT	BIO-AATAAATACTTACAAAAAACCTTCC	TGTTTGAGGATTGGGAT
RASSF1	AGTTTGGATTTTGGGGGAGG	BIO-CAACTCAATAAACTCAAACTCCCC	GGGTTAGTTTTGTGGTTT

All reverse primers are biotinylated (BIO) at their respective 5’-end

**Table 3:** PCR conditions for amplification of gene promoter regions.

Assay	Initial Denaturation	Cycles	Denaturation	Annealing	Elongation	Extension
APC	95°C / 15 min	45 x	95°C / 30s	56°C / 30s	72°C / 30s	72°C / 10min
CDH1	95°C / 15 min	45 x	95°C / 30s	59°C / 30s	72°C / 30s	72°C / 10min
EFEMP1	95°C / 15 min	45 x	95°C / 30s	59°C / 30s	72°C / 30s	72°C / 10min
FHIT	95°C / 15 min	45 x	95°C / 30s	63°C / 30s	72°C / 30s	72°C / 10min
L1RE1	95°C / 15 min	45 x	95°C / 30s	50°C / 30s	72°C / 30s	72°C / 10min
MGMT	95°C / 15 min	45 x	95°C / 30s	53°C / 30s	72°C / 30s	72°C / 10min
CDKN2A (P14)	95°C / 15 min	45 x	95°C / 30s	52°C / 30s	72°C / 30s	72°C / 10min
PTEN	95°C / 15 min	45 x	95°C / 30s	55°C / 30s	72°C / 30s	72°C / 10min
RARB	95°C / 15 min	45 x	95°C / 30s	52°C / 30s	72°C / 30s	72°C / 10min
RASSF1	95°C / 15 min	45 x	95°C / 30s	58°C / 30s	72°C / 30s	72°C / 10min

### TCGA—DNA methylation analysis

For the TCGA data a comprehensive genome-wide DNA methylation profile was generated. Therefore, a Illumina Methylation Assay (either Infinium HumanMethylation27 BeadChip or Infinium HumanMethylation450 BeadChip) was used. Similar to our approach, the method generates a methylation level profile (in percent) for the CpG dinucleotides investigated. *RASSF1* and *RARB* were available for both data sets (TCGA-LUAD and TCGA-LUSC, referenced above). *L1RE1* was not available in the database. Data for 178 ADC and 104 SQCC was available; no methylation data for benign or NET was available.

### Statistical analysis

Statistical and graphical analyses were performed using the R environment [15]. Where feasible, the Shapiro-Wilk-test was applied as test of normality. Based on the results, either a parametric or a non-parametric test was applied to detect group differences. For dichotomous variables, such as gender, invasion into lymphatic vessels and veins, and methylation level either the Wilcoxon Mann-Whitney rank sum test or students t-test (two-sided) was applied. For variables with more than two groups (e.g., tumor type, TNM staging) either the Kruskal-Wallis test or ANOVA was used to detect group differences. Correlations between methylation levels and spread to regional lymph nodes, methylation analysis between genes as well as age of the patients were tested by using the Spearman’s rank correlation test.

Due to the multiple statistical tests the p-values were adjusted by using the false discovery rate (FDR). The level of statistical significance was defined as p≤0.05 after adjustment.

A heatmap was created to visualize the results of the unsupervised hierarchical cluster analysis. Sensitivity and specificity of methylation markers were determined from receiver operating characteristic (ROC) curves illustrating their performance to discriminate the studied groups. The threshold for methylation levels was derived from the ROC analysis with a set specificity of ≥95% where possible. The bootstrap procedure (1000 runs) was used for internal validation of the estimates in the ROC analyses. Methylation profiles were explored with the Classification and Regression Tree Algorithm (CART) or Conditional Interference Trees (ctree) as implemented in the rpart library of R using a leave-one-out cross validation [16].

S1 File (exploratory set) and S2 File (validation set) summarize the data that was used for statistical analysis.

## Results

**Fig 1:**
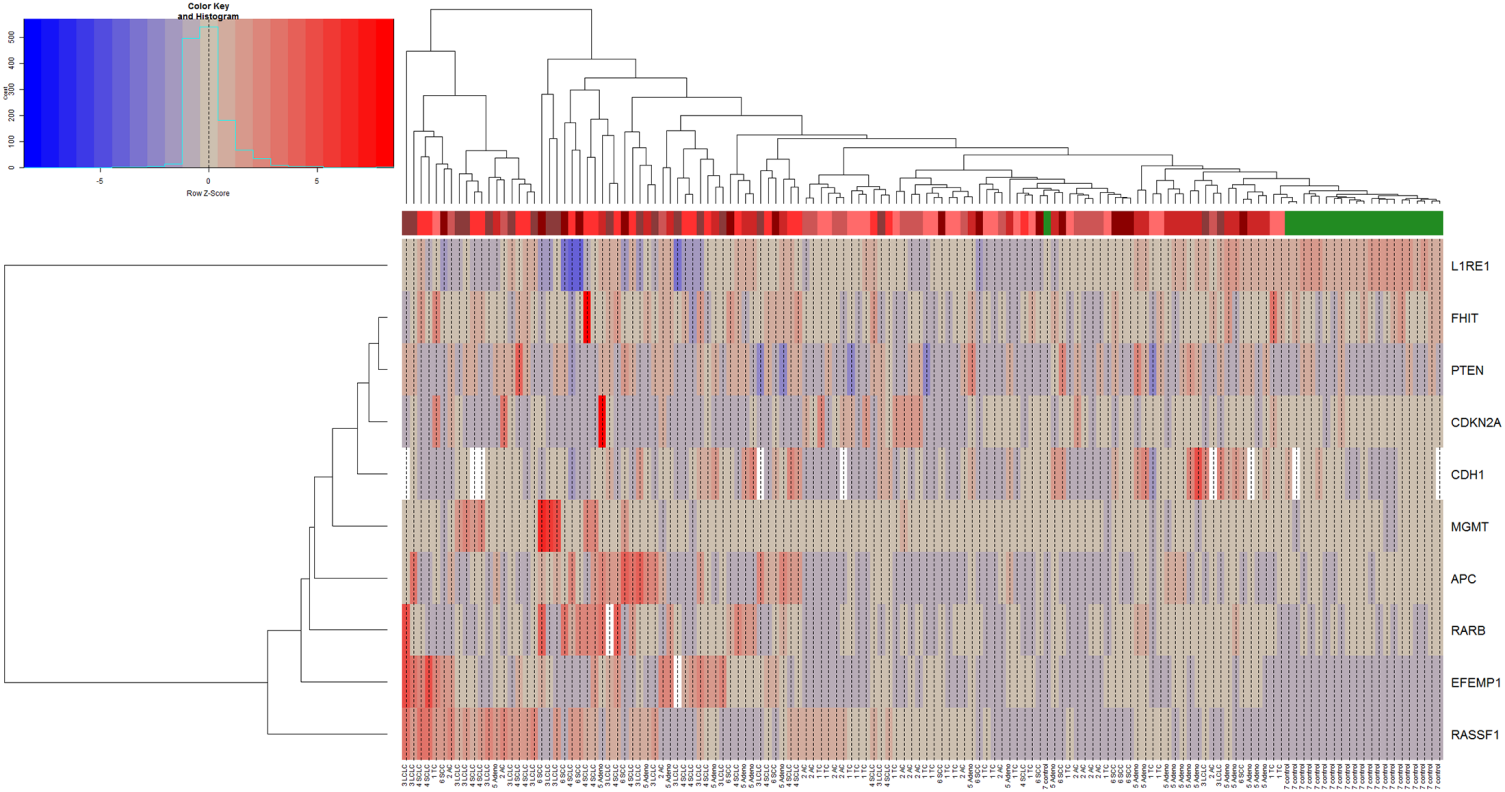
Heatmap of the methylation markers investigated and their differential methylation with respect to the investigated tissue types. On the bottom x-axis each investigated patient with its entity identifier is depicted. The upper x-axis shows the binary clusters of the entity groups with respect to the methylation markers, which are shown on the y-axis. Each tumor entity is represented by one of the red colors, whereas green represents benign tissue. The main clusters represent benign (predominantly green) versus tumor (predominantly red) samples. Benign tissue shows differential methylation compared to tumors with respect to *EFEMP1*, *L1RE1*, *RARB*, and *RASSF1*.

With few exceptions, most of the 116 tumor samples and 22 controls could be analyzed successfully with the *APC*, *CDH1*, *CDKN2A*, *EFEMP1*, *FHIT*, *L1RE1*, *MGMT*, *PTEN*, *RARB*, and *RASSF1* assays in the exploratory dataset. The assay for *EFEMP1* and *RARB* failed to show results in one patient and the *CDH1* assay failed in seven patients and two controls samples, although triplicates were tested. Several tested methylation markers showed significantly differential methylation between the investigated entities, mostly differentiating benign from tumor samples as shown in the heatmap (Fig 1).

**Fig 2:**
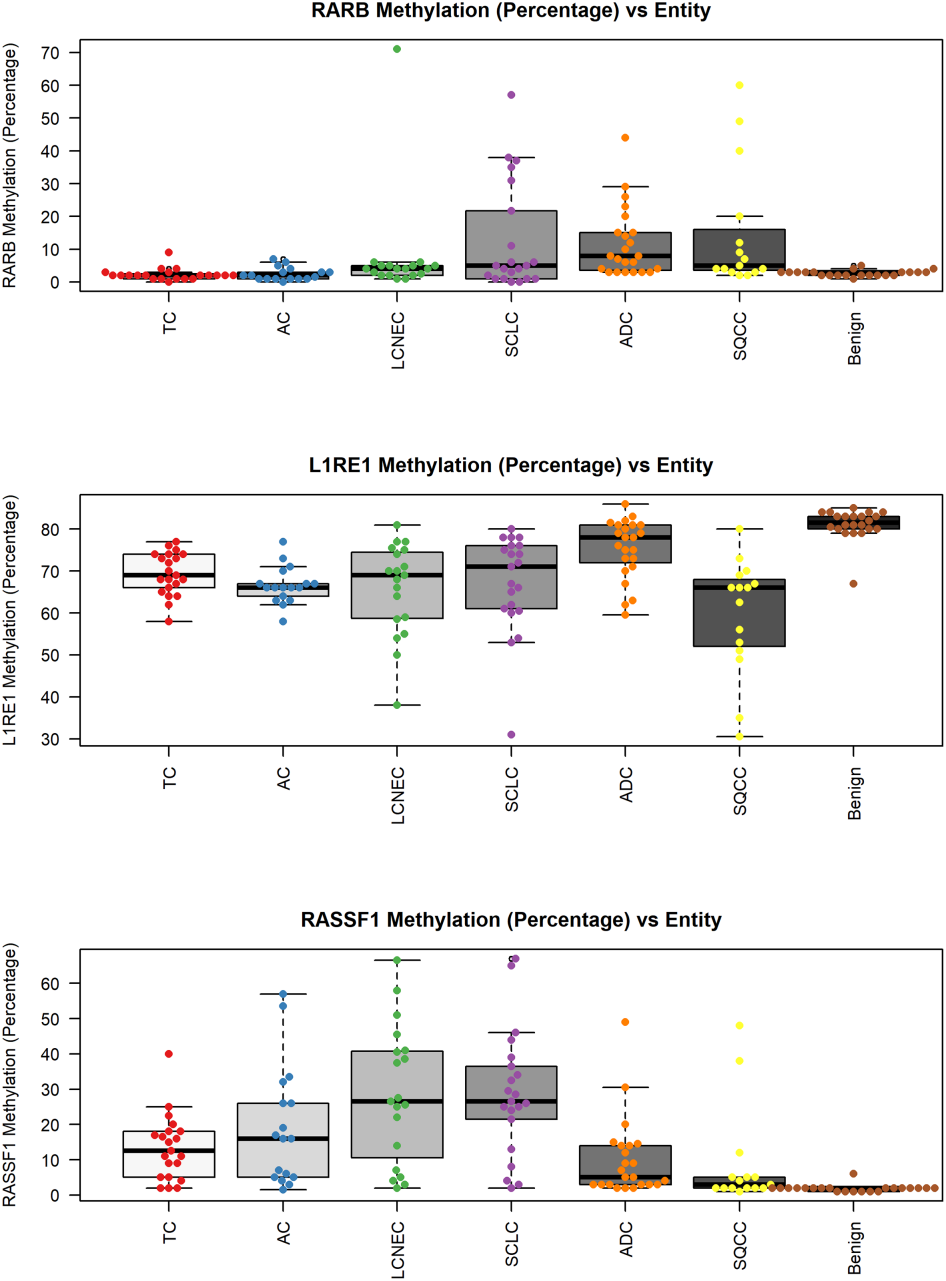
Correlation of *L1RE1*, *RASSF1*, and *RARB* methylation and tissue type. Associations between the tissue subtype and methylation level of A) *L1RE1*, B) *RASSF1*, and C) *RARB* are pictured as boxplots. On the x-axis the seven investigated tissue subtypes are depicted. The y-axis shows the methylation in percent. The p-value is based on a Kruskal-Wallis rank sum test and is rounded to the fourth decimal place. *L1RE1* methylation was lower in tumors than in benign tissue. *RASSF1* methylation was higher in tumors than in benign control tissue and methylation increased in neuroendocrine tumors of the lung from TC to SCLC. *RARB* hypermethylation was more prominent in high-grade lung cancer than in pneumothoraces and carcinoids (TC and AC).

Fig 2 shows boxplots of methylation levels of *L1RE1*, *RASSF1*, and *RARB* for the different tumor entities investigated in comparison to benign tissues that served as controls. S1 Fig shows the graphical results for the corresponding ROC analyses. *L1RE1* was able to discriminate tumor samples and controls (p<0.001, Table 4) with a sensitivity of 88.8% and a specificity of 95%, when using a threshold of <78.5% methylation, as determined by ROC analysis. *RASSF1* discriminated benign from tumor samples (p<0.001) with a sensitivity of 86.2% and specificity of 95% (threshold >2.5% methylation according to ROC analysis). The subgroup of neuroendocrine tumors (NET: TC, AC, LCNEC, and SCLC) was discriminated from benign samples by *RASSF1* with a sensitivity of 92.3% and a specificity of 95% (p<0.001, threshold >2.5% methylation according to ROC analysis). High-grade NET (LCNEC and SCLC) could be separated from ADC as well as SQCC with a sensitivity of 72.5% and a specificity of 89.5% at a threshold of >14.25% methylation. For overall discrimination between tumor and benign tissue *RARB* (p<0.001) showed a sensitivity of 39.1% and specificity of 95% according to ROC analysis with a threshold >4.5%.

**Table 4:** Statistical tests applied and significances for the parameters investigated.

**Methylation level in percent**	**Gene**	**Mean**	**Median**	
	RARB	8.6	4	
	MGMT	5.6	3	
	L1RE1	67.9	69	
	CDH1	5.9	5	
	RASSF1	18.2	14	
	PTEN	1.4	1	
	CDKN2A	3.2	2.25	
	FHIT	2.2	2	
	APC	8.2	3	
	EFEMP1	12.0	7	
**Tested Parameter**	**Gene**	**p-value**	**FDR-adjusted p value**	**rho**
**Gender**	CDKN2A	0.0037	0.037	
**Spread to Regional Lymph Nodes**	**Gene**	**p-value**	**FDR-adjusted p value**	**rho**
	EFEMP1	0.0027	0.0270	0.31
	FHIT	0.0055	0.0275	0.29
	CDKN2A	0.0150	0.0500	-0.25
**Age at Diagnosis**	**Gene**	**p-value**	**FDR-adjusted p value**	**rho**
	RASSF1	0.0002	0.0015	-0.35
	CDH1	0.0029	0.0145	0.29
**Gene X Methylation vs. Gene Y Methylation**	**Gene**	**p-value**	**FDR-adjusted p value**	**rho**
	MGMT vs. RASSF1	<0.0001	<0.0001	0.41
	CDH1 vs. PTEN	<0.0001	0.0002	0.38
	L1RE1 vs. RASSF1	<0.0001	0.0008	-0.34
	RARB vs. CDH1	0.0004	0.0046	0.31
	RASSF1 vs. EFEMP1	0.0005	0.0046	0.29
	L1RE1 vs. CDH1	0.0009	0.0055	0.29
	L1RE1 vs. CDKN2A	0.0008	0.0055	0.28
	CDH1 vs. RASSF1	0.0015	0.0084	-0.28
	RASSF1 vs. CDKN2A	0.0022	0.0110	-0.26
	MGMT vs. L1RE1	0.0034	0.0153	-0.25
	CDKN2A vs. EFEMP1	0.0038	0.0155	-0.25
	MGMT vs. PTEN	0.0060	0.0225	0.23
	RARB vs. APC	0.0074	0.0256	0.23
	CDH1 vs. EFEMP1	0.0089	0.0286	0.23
	CDH1 vs. APC	0.0120	0.0360	0.22
	RARB vs. PTEN	0.0150	0.0422	0.21
	RARB vs. CDKN2A	0.020	0.0500	-0.2
	FHIT vs. APC	0.019	0.0500	0.2
**Grade of the Tumor**	**Gene**	**p-value**	**FDR-adjusted p value**	**rho**
	RASSF1	<0.0001	<0.0001	0.55
	CDKN2A	<0.0001	<0.0001	-0.5
	EFEMP1	<0.0001	<0.0001	0.51
	RARB	<0.0001	<0.0001	0.42
	L1RE1	<0.0001	<0.0001	-0.37
	MGMT	0.0001	0.0002	0.34
	PTEN	0.0007	0.0010	0.3
	APC	0.0380	0.0475	0.19
**Histological Subtype**	**Gene**	**p-Value**	**FDR-adjusted p value**	
	RASSF1	<0.0001	<0.0001	
	L1RE1	<0.0001	<0.0001	
	CDH1	<0.0001	<0.0001	
	CDKN2A	<0.0001	<0.0001	
	EFEMP1	<0.0001	<0.0001	
	RARB	<0.0001	<0.0001	
	MGMT	<0.0001	0.0011	
	PTEN	0.0036	0.0045	
	APC	0.0041	0.0046	
	FHIT	0.0220	0.0220	

**Fig 3:**
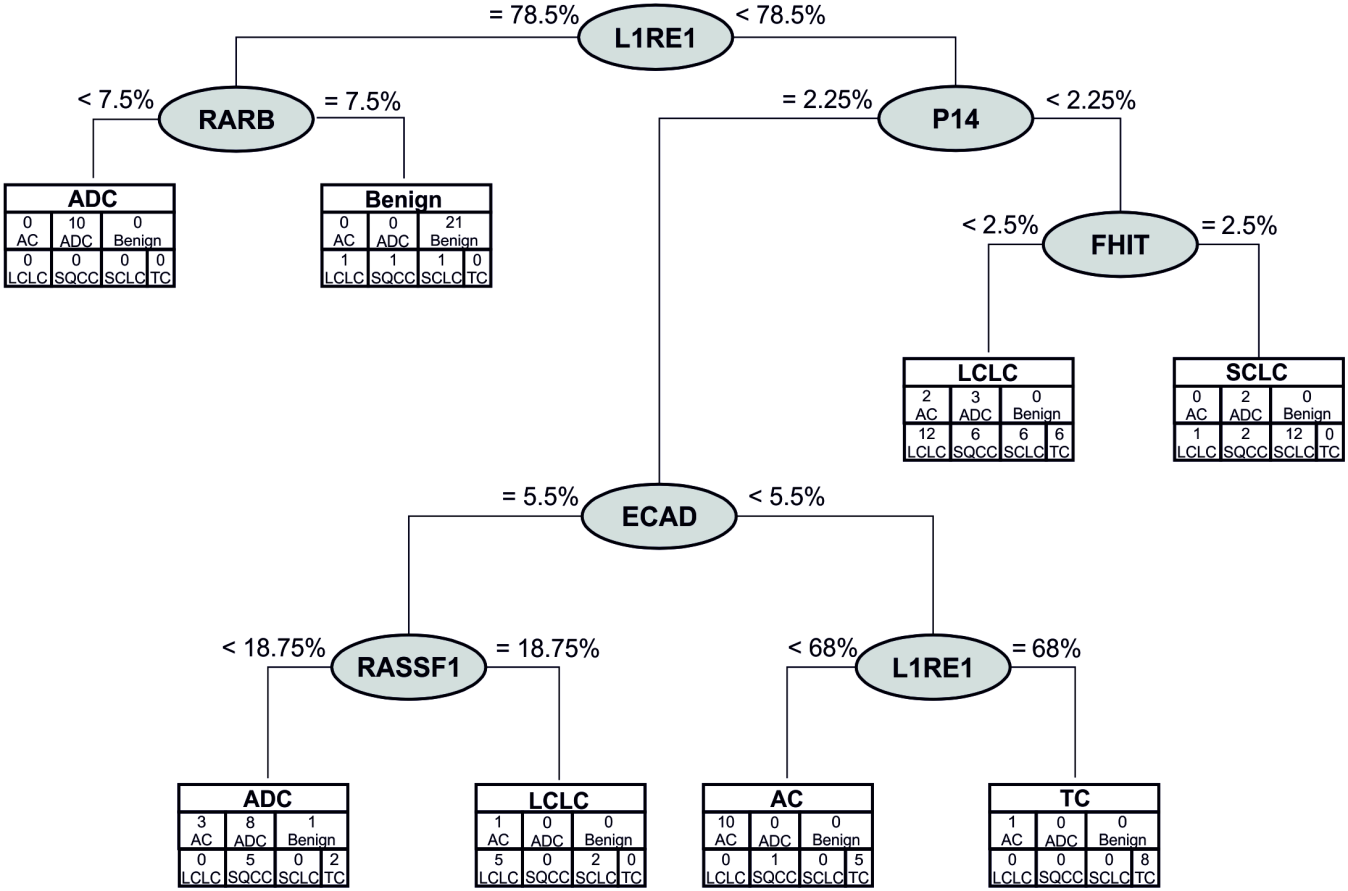
CART analysis of investigated methylation markers. *CDKN2A (P14)*, *CDH1*, *FHIT*, *L1RE1*, *RASSF1*, and *RARB* separated the tumor subtypes and benign samples with a failure rate of 51%. Higher *L1RE1* and low *RARB* methylation discriminated benign from tumor samples. SQCC was not discriminated from the other subtypes.

Fig 3 depicts the CART classification of lung cancer in all samples by histological subtype according to all investigated methylation markers. *L1RE1*, *RARB*, *CDKN2A (P14)*, *CDH1*, *RASSF1*, and *FHIT* separated the subtypes in seven nodes with a failure rate of 51%. Higher *L1RE1* and low *RARB* methylation discriminated benign from tumor samples. However, SQCC was not discriminated from the other subtypes.

**Fig 4:**
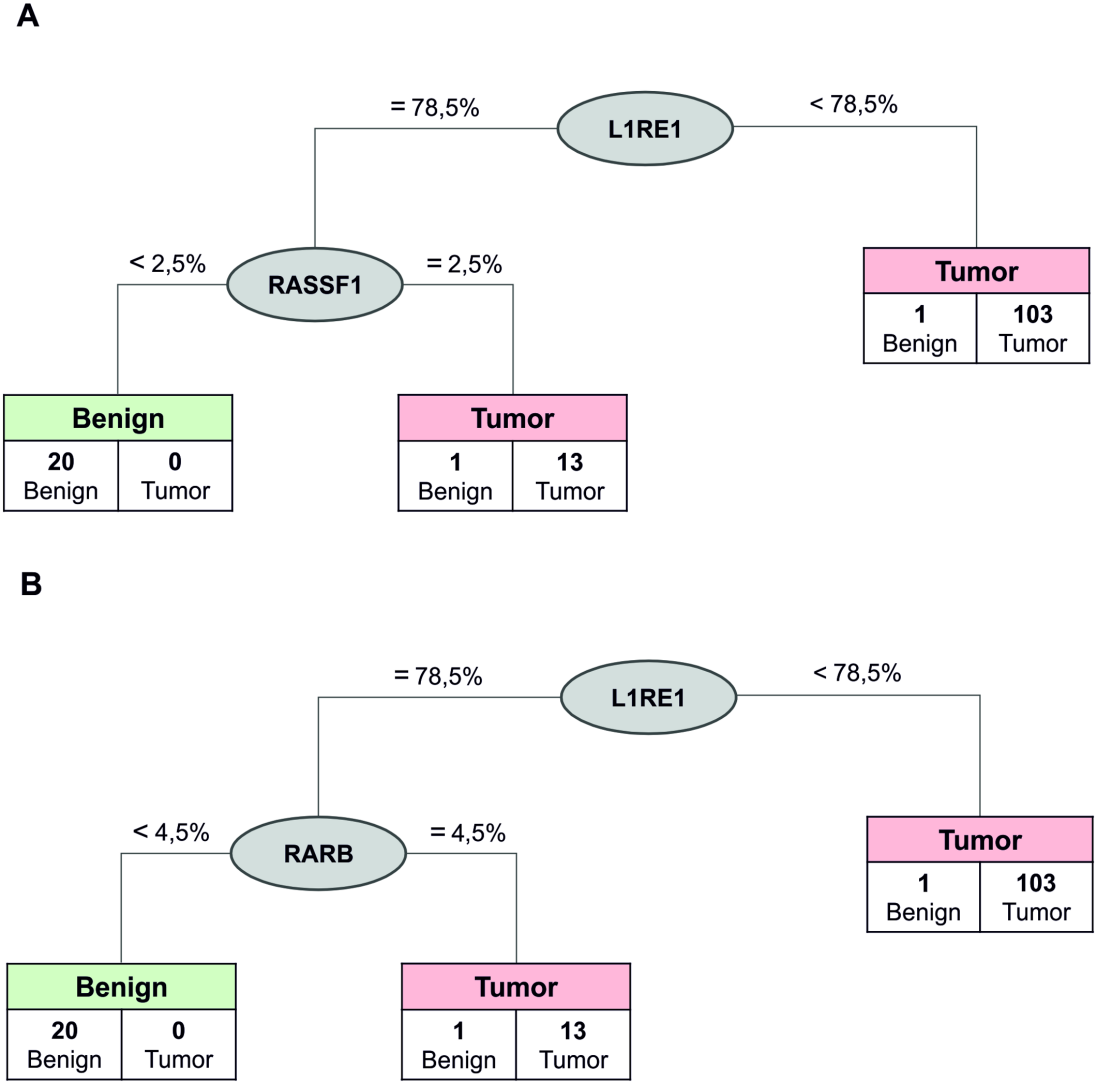
Separation of tumor and benign tissue by *L1RE1* and *RASSF1* or *L1RE1* and *RARB*. CART analysis of two-marker combinations to discriminate lung tumors and benign tissues. A) Using a combination of *L1RE1* and *RASSF1* all but two benign samples were separated from tumor tissue when considering a *L1RE1* methylation ≥78.5% and a *RASSF1* methylation <2.5%. The corresponding sensitivity and negative predictive value were 100%, the specificity 90.0%, and the positive predictive value 98.3%. The failure rate was 1.4%. B) The combination of *L1RE1* and *RARB* showed a performance similar to that of *L1RE1* and *RASSF1*.

Furthermore, according to the CART results the combination of *L1RE1* and *RARB* was able to separate tumor and benign tissue with a failure rate of 1.4% (91% specificity and 100% sensitivity). *L1RE1* and *RASSF1* separated control samples from tumor samples in the same way (Fig 4). In both trees, lower *L1RE1* methylation discriminated most tumor samples from ADC and benign samples. In the second node, higher methylation of either *RASSF1* or *RARB* was able to separate the remaining tumor from the benign samples (data not shown). However, two benign samples were classified as tumor.

Age of the patients, gender, and additional pathological parameters showed no influence on *L1RE1* and *RARB* methylation (data not shown). Of note, *RASSF1* correlated inversely with age of the patients (p = 0.0015, Table 4).

Significant correlations of methylation levels with tumor grade were found for *APC*, *CDKN2A*, *EFEMP1*, *L1RE1*, *MGMT*, *PTEN*, *RARB*, and *RASSF1*. *CDKN2A* and *PTEN* showed minimal to absent methylation, therefore, an analysis regarding tumor grade was deemed to be not meaningful (data not shown). For the remaining methylation markers, the results with respect to tumor grade are depicted in S2 Fig. In particular, *RASSF1* showed a pronounced increase of methylation frequency with tumor grade.

Spread to regional lymph nodes correlated significantly with increasing methylation of *EFEMP1* (p = 0.027) and *FHIT* (p = 0.028) with a linear dependence, whereas an inverse correlation was found for *CDKN2A* methylation (p = 0.05). However, *CDKN2A* and *FHIT* showed minimal to absent methylation (data not shown). The result for *EFEMP1* is shown in S3 Fig. The statistically significant results can be found in Table 4.

### Validation of the results

Within the validation dataset (27 lung cancer cases and 38 benign controls) *L1RE1*, *RARB*, and *RASSF1* were analyzed successfully. From the TCGA database, 282 lung cancer (178 ADC and 104 SQCC) samples were also analyzable for methylation of *RASSF1* and *RARB*. *L1RE1* was not available for TCGA data sets. In addition, no data for NET or benign samples was available.

**Fig 5:**
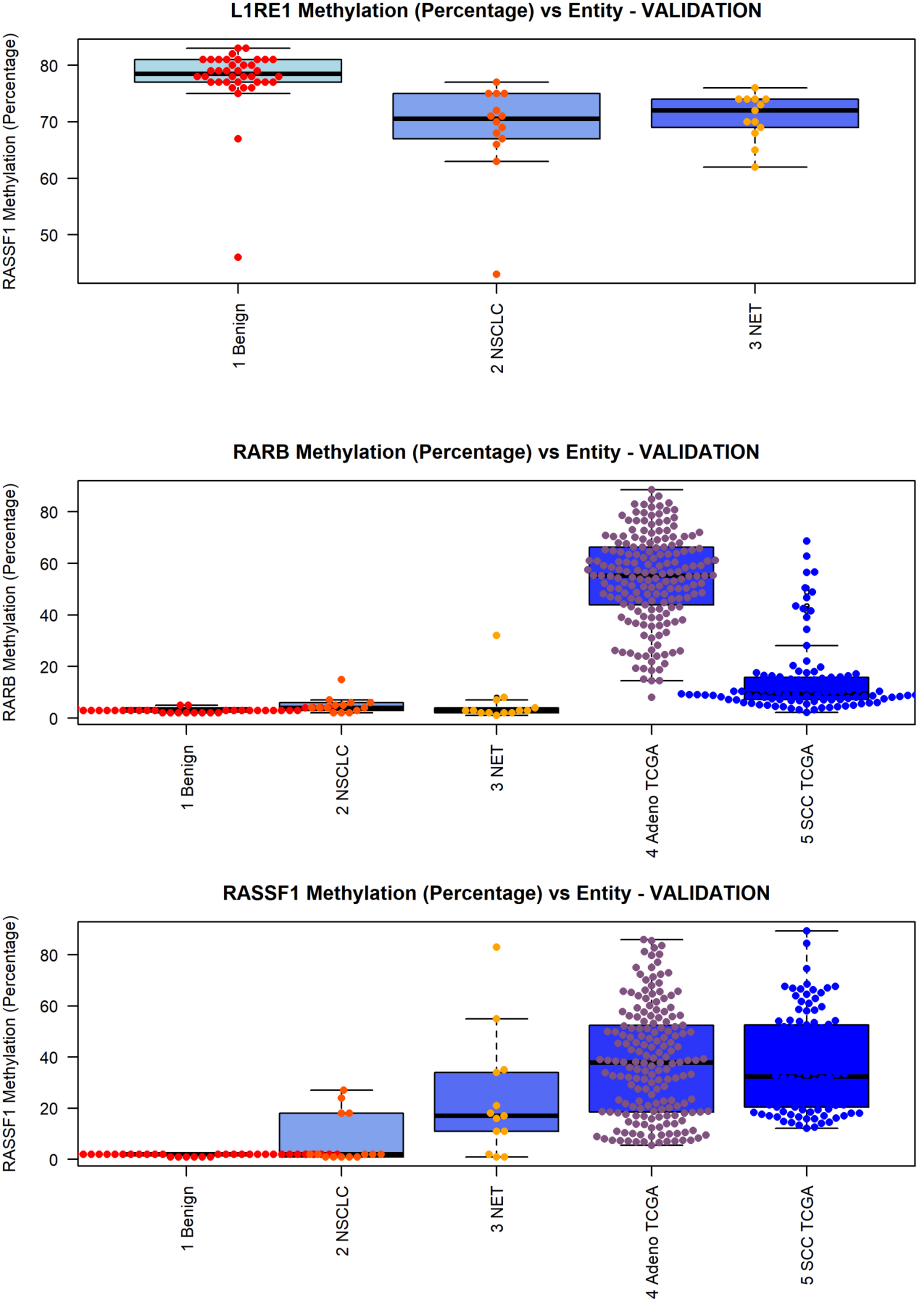
Correlation of *L1RE1*, *RASSF1*, and *RARB* methylation and tissue type. Associations between the tissue subtype and methylation level of A) *L1RE1*, B) *RASSF1*, and C) *RARB* are pictured as boxplots. On the x-axis the investigated tissue subtypes are depicted. The y-axis shows the methylation in percent. The p-value is based on a Kruskal-Wallis rank sum test and is rounded to the fourth decimal place. *L1RE1* methylation was lower in tumors than in benign tissue. *L1RE1* was not available for the TCGA data. *RASSF1* methylation was higher in tumors than in benign control tissue. Elevated *RARB* methylation was more prominent in lung cancer than in benign samples.

The validation dataset confirmed the above presented results. *L1RE1* hypermethylation was more prominent in benign compared to lung cancer samples. *RARB* showed nearly absent methylation in benign samples; cancer samples showed slight methylation. TCGA data showed differential methylation in ADC and SQCC. For *RASSF1* the previous results were strongly confirmed: Benign samples showed no methylation, whereas cancer samples present with elevated methylation. Fig 5 depicts the results for the validation dataset and TCGA data. Table 5 summarizes the results of the statistical tests.

**Table 5:** Statistical tests applied and significances for the parameters investigated.

**Methylation level in percent**	**Gene**	**Mean**	**Median**
**For the validation dataset**	RARB	4	3
	L1RE1	74	77
	RASSF1	7	4
**For TCGA data**	RARB	44	40
	RASSF1	35	37
**Histological Subtype**	**Gene**	**p-Value**	**FDR-adjusted p value**
**For the validation dataset**	RARB	0.058	0.07
	L1RE1	<0.0001	<0.0001
	RASSF1	0.0009	0.0018

**Fig 6:**
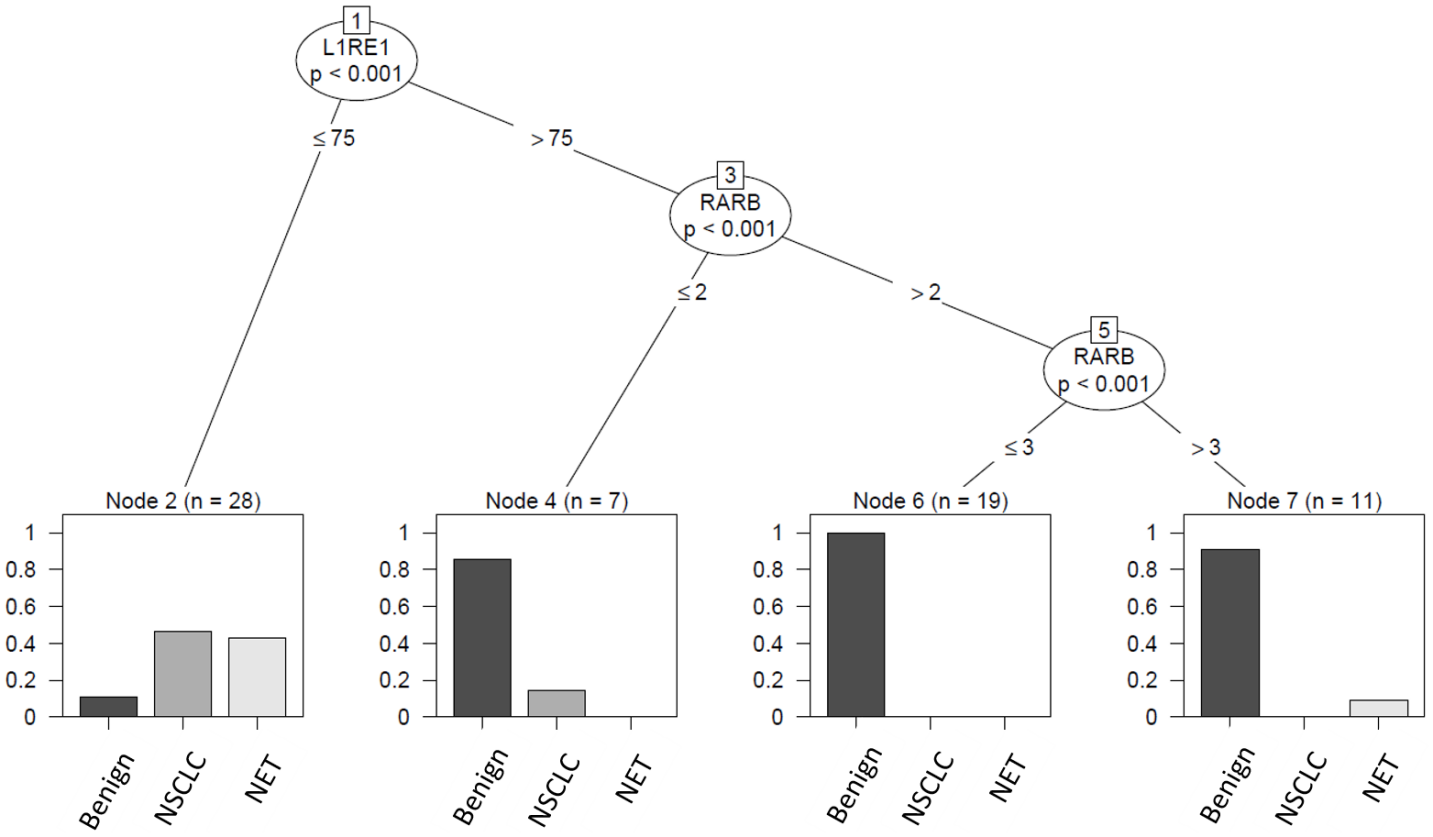
Separation of tumor and benign tissue *L1RE1* and *RARB*. Decision tree analysis confirmed previous findings: *L1RE1* and *RARB* were able to discriminate benign from cancer samples with similar resolution as in the exploratory set.

Fig 6 depicts the results for the decision tree calculation for *L1RE1* and *RARB*. Both methylation markers in combination were able to separate tumor and benign tissue in the validation dataset with comparable resolution as found for the exploratory set.

## Discussion

Here we present the results of 116 lung tumor samples of different histology and 22 benign controls and their methylation profile of ten potential methylation biomarkers, which were investigated by pyrosequencing. CART classification failed to discriminate lung cancer by histological subtype according the analyzed methylation markers. However, combinations of two methylation biomarkers, i.e. *L1RE1* and *RARB* or *L1RE1* and *RASSF1*, were sufficient to discriminate tumor from benign tissue with 91% specificity and 100% sensitivity using CART classification. A good separation between tumor and benign tissue was also demonstrated by an independent method, unsupervised hierarchical clustering. Similar to the CART classification, a further discrimination of histological subtypes based on the chosen markers was not possible. However, *RASSF1* seems to be a suitable marker for the discrimination of LCNEC as well as SCLC from ADC and SQCC (Fig 2).

Hypomethylation of *L1RE1* (also known as *LINE1*) was identified as a marker for aggressiveness in several cancers [8], which is confirmed by our data. Recent investigations report a MILI-LINE1-Magea pathway that regulates the methylation of transposons, especially *L1RE1* [8]. *L1RE1* is one of the most common transposons in mammals and has a major impact on gene organization and gene expression depending on its methylation status, and aberrant methylation is associated with higher genetic instability and more aggressive behavior of the respective tumor [8, 9]. *L1RE1* can be considered as a global methylation marker as it comprises approximately 17% of the human genome [9]. With respect to our results, hypomethylation (≤78.5% methylation) of *L1RE1* is frequent in lung cancer and can separate tumor from benign tissue with high specificity (95.5%) and sensitivity (88.8%), using CART.

Promoter hypermethylation of *RASSF1* was reported as a frequent event in lung cancer and the highest methylation (up to 100%) was found in SCLC [7]. In our study, *RASSF1* hypermethylation was mainly observed in NET of the lung and increased from low-grade TC to high-grade SCLC. Interestingly, in the thyroid, another endocrine organ, *RASSF1* promoter methylation was observed as an early event in follicular thyroid hyperplasia and it was suggested that it paves the way for thyroid tumorigenesis [6] indicating a mechanism that might be specific for (neuro-)endocrine tumors. In contrast to Pelosi et al. [13] we found that there is a direct correlation between *RASSF1* promoter methylation and tumor grade. In line with their findings [13], we can confirm that methylation was absent in benign tissue. Besides, their results [13] and ours indicate *RASSF1* hypermethylation to be specific for NET of the lung.

*RARB* is an important tumor suppressor and loss of expression is associated with uncontrolled tumor growth and evasion of apoptosis in several tissues [5]. Furthermore, hypermethylation of *RARB* is linked to chemoresistance, therefore a clinical trial tested isotretinoin (13-cis-retinoic acid) as a therapeutic drug [5]. High *RARB* methylation may lead to lowered RARB expression sensitizing tumors for therapy with isotretinoin. Isotretinoin was tested in comparison to a placebo and it was found that risk for second primary cancers was reduced significantly after 32 months of follow-up in the isotretinoin-arm as compared to placebo, indicating that isotretinoin could be a potent drug in these tumors [5]. In our study, increasing *RARB* methylation was associated with more aggressive subtypes of lung cancer and also increased with tumor grade (both p<0.001). Inactivation of the tumor suppressors *RARB* and *CDKN2A* was identified as crucial hallmark of tumor development and maintenance in distinct tumor types [12, 17] and loss of *CDKN2A* is expected to have a similar effect as loss of *TP53* correlating with more aggressive tumors [17]. In the present study, *CDKN2A* methylation correlated with lymph node invasion, but overall methylation of *CDKN2A* was only minimal. Similar methylation patterns were found for *EFEMP1*, which showed higher methylation in tumors with higher degree of spread to lymph nodes.

*MGMT* is a controversially discussed methylation biomarker and a recent meta-analysis by Chen et al. assessed its value as biomarker with respect to staging and prognosis [18]. In line with their results, *MGMT* only correlated with tumor grade and tumor type. However, *MGMT* did not allow a clear discrimination of the tumor type and hence might be limited as a biomarker in lung cancer.

Abnormally elevated DNA concentrations in blood samples of patients with tumors were reported and could be successfully used for methylation analysis [19]. Current investigations used methylation biomarkers in serum samples for the successful discrimination of multiple pulmonary diseases such as tumors, COPD, and fibrotic ILD, and were able to separate tumor and benign samples with a sensitivity of 87.8% and specificity of 90.2% [20]. Based on our results *L1RE1* and *RARB* as well as *L1RE1* and *RASSF1* might serve as biomarkers for less invasive investigations such as liquid-biopsies or tumor biopsies (e.g., derived by endobronchial ultrasound transbronchial needle aspiration (*EBUS*-*TBNA*)). Because some of our samples were already derived from biopsies, small tissue samples do not appear to be a limiting factor. However, as tumor DNA in blood is likely to be diluted, the high specificity and sensitivity obtained for tissue is expected to be reduced in liquid biopsies. In addition to the discrimination between tumor and tumor-free samples, *RASSF1* might be a suitable marker to differentiate high-grade NET from NSCLC.

Recent investigations of epigenetic changes indicate that technical approaches, collectives investigated, and tested gene loci lead to controversial results with respect to significances and clinically relevant results [7, 11, 17]. Hence, the TCGA data set and our exploratory and validation cohorts showed different results. The TCGA data is based on a BeadChip technology, used for screening studies. As expected, our validation cohort showed similar results compared to the exploratory cohort. But, further investigations are needed and method specific results can be expected. This should also include the evaluation of the epigenetic biomarker candidates in easily accessible body fluids for early detection of cancer. For this evaluation, however, cross-sectional or case-control studies are not suitable [21]. Instead, the biomarkers would have to be validated in longitudinal studies using prediagnostic samples.

In conclusion, we have confirmed that aberrant promoter methylations of *RASSF1*, *L1RE1*, and *RARB* have the potential to separate lung tumors from benign tissue but are limited in their ability to diagnose specific subtypes. Regarding staging and grading, *RASSF1* and *EFEMP1* might offer some benefit. The analyses were not limited by small sample sizes. Using biomarkers as a minimally-invasive tool requires detectability in easily accessible body fluids. The next step, therefore, would be to test the markers in plasma samples.

## Supporting information

S1 Fig ROC analysis of *L1RE1*, *RASSF1*, and *RARB* for the differentiation of tumor versus benign samples. The ROC curves display the sensitivity and specificity of the tested methylation markers for their discriminative power between tumor and benign samples. On the x-axis the specificity is plotted. The y-axis shows the sensitivity. Area under curve (AUC) with 95% confidence interval (CI) was calculated and is included in the plot. For A) *L1RE1* a sensitivity of 88.8% and specificity of 95% with a cut-off at 78.5% methylation can be derived. For B) *RASSF1* a cut-off at 2.5% methylation results in a sensitivity of 86.2% and a specificity of 95%. For C) *RARB* methylation (4.5% as cut-off) a sensitivity of 39.1% and specificity of 95% can be derived from the ROC curve.
(TIF)

S2 Fig Correlation of *APC*, *EFEMP1*, *L1RE1*, *MGMT*, *RASSF1*, and *RARB* methylation and grade of the tumor. Associations between methylation levels of A) *L1RE1*, B) *RARB*, C) *RASSF1*, D) *MGMT*, E) *APC*, F) *EFEMP1* and grade of the tumor are pictured as boxplots. On the x-axis the grade of the tumor and benign controls are depicted. The y-axis shows methylation in percent. Samples with unknown grade were excluded. The p-value is based on a Spearman’s rho test and is rounded to the fourth decimal place. A direct linear correlation between increasing methylation and higher grade was found for *EFEMP1*, *MGMT*, *RASSF1*, and *RARB*. An inverse correlation was not found. *L1RE1* methylation decreased between benign and tumor regardless of the tumor grade. *APC* was unable to differentiate benign from tumor grade (no linear correlation), but increased with higher grade.
(TIFF)

S3 Fig Correlation of *EFEMP1* methylation and degree of spread to regional lymph nodes. The association between the methylation level of *EFEMP1* and lymph node invasion is pictured as boxplots. On the x-axis the degree of spread to regional lymph nodes is depicted. Samples with unknown status were excluded from the graphs. The y-axis shows methylation in percent. The p-value is based on a Spearman’s rho test and is rounded to the fourth decimal place. A direct correlation between increasing methylation and higher degree of spread was found, although N2 status presented with similar methylation as node-negative samples.
(TIFF)

S1 File Exploratory dataset used for statistical analysis. (TXT)

S2 File Validation dataset used for statistical analysis. (TXT)

S1 Table Investigated samples for validation purposes (validation dataset and TCGA data). (DOCX)

## Abbreviations

AC: atypical carcinoid tumor
ADC: adenocarcinoma
AUC: area under curve
CART: classification and regression tree algorithm
FFPE: formalin-fixed, paraffin-embedded
LCNEC: large cell neuroendocrine tumors
NET: neuroendocrine tumor
NSCLC: non-small cell lung carcinoma
ROC: receiver operating characteristic
SCLC: small cell lung carcinoma
SQCC: squamous cell carcinoma
TC: typical carcinoid tumor
TCGA: The Cancer Genome Atlas
