# Supplementary figures and images for: Methylation of L1RE1, RARB, and RASSF1 function as possible biomarkers for the differential diagnosis of lung cancer
Source: PLoS One. 2018 May 31;13(5):e0195716. doi: 10.1371/journal.pone.0195716 (PMC5978787; doi:10.1371/journal.pone.0195716)

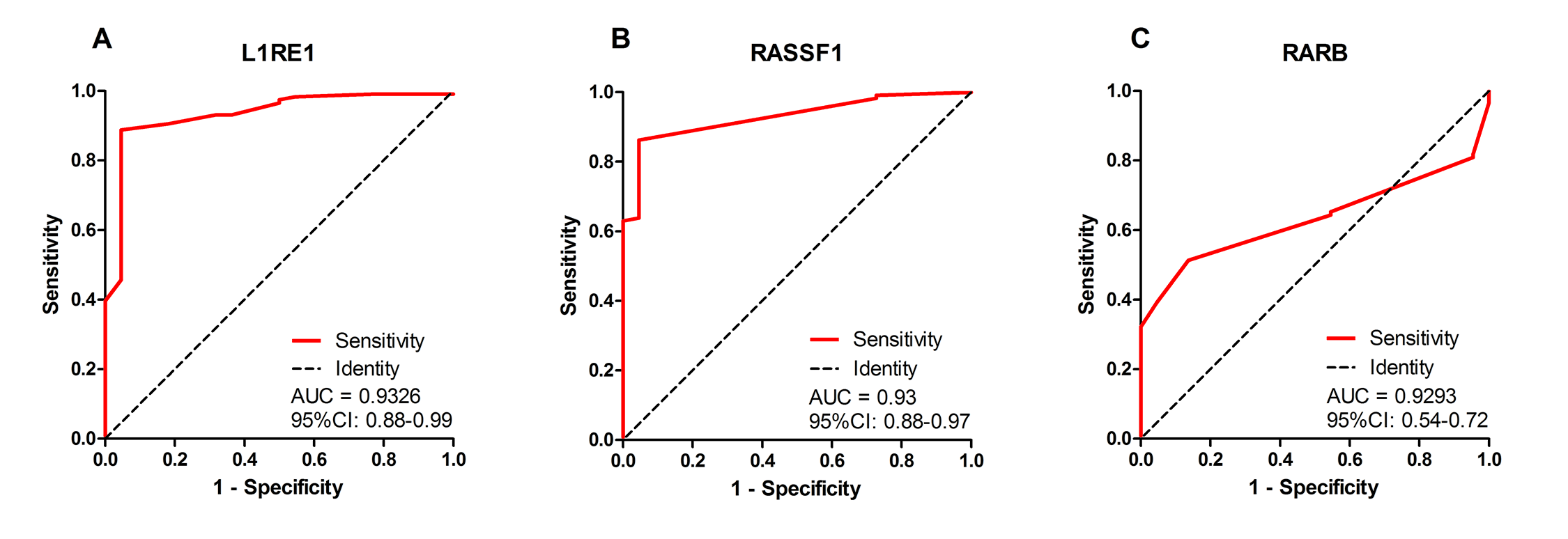

Supplement: S1 Fig — The ROC curves display the sensitivity and specificity of the tested methylation markers for their discriminative power between tumor and benign samples. On the x-axis the specificity is plotted. The y-axis shows the sensitivity. Area under curve (AUC) with 95% confidence interval (CI) was calculated and is included in the plot. For A) L1RE1 a sensitivity of 88.8% and specificity of 95% with a cut-off at 78.5% methylation can be derived. For B) RASSF1 a cut-off at 2.5% methylation results in a sensitivity of 86.2% and a specificity of 95%. For C) RARB methylation (4.5% as cut-off) a sensitivity of 39.1% and specificity of 95% can be derived from the ROC curve. (TIF) [file pone.0195716.s001.tif]

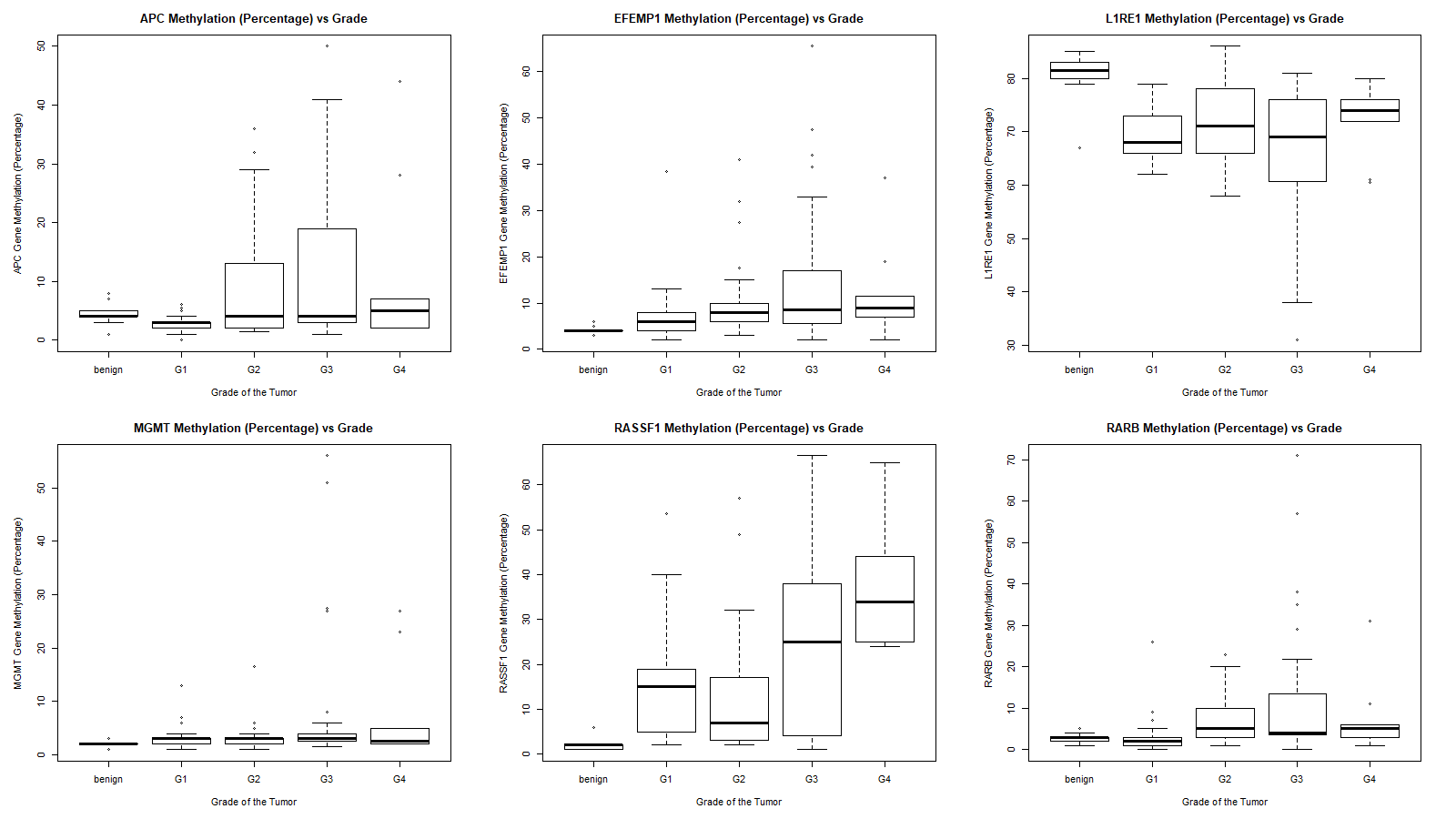

Supplement: S2 Fig — Associations between methylation levels of A) L1RE1, B) RARB, C) RASSF1, D) MGMT, E) APC, F) EFEMP1 and grade of the tumor are pictured as boxplots. On the x-axis the grade of the tumor and benign controls are depicted. The y-axis shows methylation in percent. Samples with unknown grade were excluded. The p-value is based on a Spearman’s rho test and is rounded to the fourth decimal place. A direct linear correlation between increasing methylation and higher grade was found for EFEMP1, MGMT, RASSF1, and RARB. An inverse correlation was not found. L1RE1 methylation decreased between benign and tumor regardless of the tumor grade. APC was unable to differentiate benign from tumor grade (no linear correlation), but increased with higher grade. (TIFF) [file pone.0195716.s002.tiff]

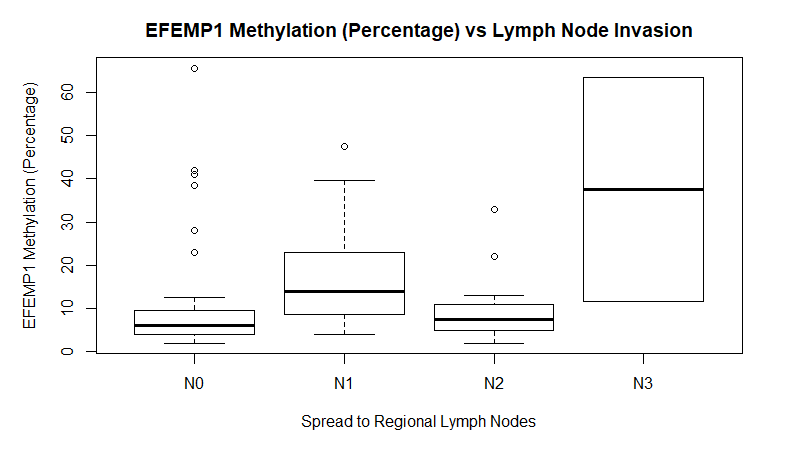

Supplement: S3 Fig — The association between the methylation level of EFEMP1 and lymph node invasion is pictured as boxplots. On the x-axis the degree of spread to regional lymph nodes is depicted. Samples with unknown status were excluded from the graphs. The y-axis shows methylation in percent. The p-value is based on a Spearman’s rho test and is rounded to the fourth decimal place. A direct correlation between increasing methylation and higher degree of spread was found, although N2 status presented with similar methylation as node-negative samples. (TIFF) [file pone.0195716.s003.tiff]
