# Supplementary material for: Methylation of L1RE1, RARB, and RASSF1 function as possible biomarkers for the differential diagnosis of lung cancer
Source: PLoS One. 2018 May 31;13(5):e0195716. doi: 10.1371/journal.pone.0195716 (PMC5978787; doi:10.1371/journal.pone.0195716)
Supplement: S1 Table — (DOCX) [file pone.0195716.s006.docx]

|  | **Validation cohort** |  |  | **TCGA-derived cohort** |
| --- | --- | --- | --- | --- |
|  | **N** |  |  | **N** |
| **Benign** | 38 |  | **Benign** | none |
| **Lung Cancer** | 27 |  | **Lung Cancer** | 282 |
| **NSCLC** | 14 |  | **ADC (NSCLC)** | 178 |
| **NET** | 13 |  | **SQCC (NSCLC)** | 104 |
